# The Effect of a Community Health Worker Utilized Mobile Health Application on Maternal Health Knowledge and Behavior: A Quasi-Experimental Study

**DOI:** 10.3389/fpubh.2018.00133

**Published:** 2018-05-07

**Authors:** Onaedo Ilozumba, Sara Van Belle, Marjolein Dieleman, Loan Liem, Murari Choudhury, Jacqueline E. W. Broerse

**Affiliations:** ^1^Faculty of Sciences, VU University Amsterdam, Amsterdam, Netherlands; ^2^Department of Public Health, Instituut voor Tropische Geneeskunde, Antwerpen, Belgium; ^3^SIMAVI, Amsterdam, Netherlands; ^4^NEEDS, Deoghar, India

**Keywords:** mobile health, community health workers, maternal health knowledge, antenatal care, institutional delivery, India, rural populations

## Abstract

**Background:**

Mobile technology (mHealth) is increasingly being used to achieve improved access and quality of maternal care, particularly in rural areas of low- and middle-income countries. In 2011, a mobile application—Mobile for Mothers (MfM)—was implemented in Jharkhand, India to support home visits by community health workers. The objective of this study is to assess the impact of the mHealth intervention on maternal health.

**Methods:**

Households from three subdistricts in the Deoghar district of Jharkhand were selected using a multistage cluster sampling approach. Households from the Sarwan subdistrict received the MfM intervention, those from Devipur subdistrict received other interventions asides MfM from the implementing non-governmental organization (NGO), while households from Mohanpur subdistrict received the current standard of care. Women (*n* = 2,200) between the ages of 18 and 45 who had delivered a baby in the past 1 year were enrolled into the study. The primary outcomes of interest were maternal health knowledge, antenatal care (ANC) attendance, and delivery in a health facility.

**Results:**

Post-intervention, women in the MfM group had higher maternal health knowledge, were more likely to attend four or more ANC visits, and deliver at the health facility when compared with the NGO and standard care group. After controlling for predictors, women in the intervention group significantly performed better than both the NGO and standard care groups on all three-outcome variables (all *P* > 0.05).

**Conclusion:**

The results indicate that although the MfM mHealth intervention could influence adherence and practice of recommended maternal health behaviors, it could not overcome key sociocultural determinants of maternal health such as caste and educational status, which are specific to the Indian context. mHealth holds continued promise for maternal health but implementers and policy makers must additionally address health system and sociocultural factors that play a significant role in the uptake of recommended maternal health practices.

## Introduction

Over the last decade, the ownership of mobile phones in low- and middle-income countries (LMICs) has significantly increased and influenced numerous domains (1–3). It is estimated that about 95% of the world’s population is currently connected to a mobile network (2). This increased availability of mobile phones and their relative ease of use have led to the development of the field of mobile health (mHealth). “mHealth” is defined as a medical and public health practice supported by mobile devices, such as mobile phones, patient monitoring devices, personal digital assistants, and other wireless devices (4). In LMICs, many mHealth interventions have been developed for the domain of maternal health care with applications for antenatal care (ANC), delivery, and postnatal care (5). Despite improvements in maternal mortality since 1990, including a 45% reduction in maternal mortality and 71% of births globally attended by a health professional, maternal mortality still remains high at an estimated 289,000 deaths annually, with LMIC accounting for approximately 99% of all maternal deaths (6). mHealth has generated much interest as a possible solution to overcoming many of the recognized barriers to maternal health care in LMICs including poor infrastructure, lack of electricity, the shortage of skilled health-care workers, remote communities, and the distance to services (4). The World Health Organization (WHO) recommendations to achieve further maternal mortality reduction include the following: four or more ANC visits during pregnancy, a skilled birth attendant at delivery, and access to postnatal care (6, 7). Achieving these WHO recommended guidelines are frequently the focus of maternal mHealth interventions.

“Mobile health” interventions are often utilized for client education and behavior change, data collection and reporting, and human resource management (8). Maternal mHealth interventions in LMICs have routinely targeted two groups of end users: health workers and pregnant women or new mothers. The inclusion of community health workers (CHWs) in these interventions is motivated by the fact that in many health systems, CHWs form an integral part of the health system, delivering maternal, and child health services at community level (and form the liaison with the pregnant women or the communities) (9–11). There is evidence in the literature for the effectiveness of mHealth interventions in increasing the utilization of maternal services by pregnant women and improvement in their emotional well-being (12–15). However, most of this evidence comes from the utilization of unidirectional text messages, i.e., women receive a text message on their phones and do not reply (15). Studies on the use of mHealth applications by CHW are focused on Sub-Saharan Africa and utilize unidirectional or multidirectional text messaging, i.e., women can respond to messages they receive (16). There is, however, less known about the effectiveness of mobile applications, utilized by CHWs during home visits on the health outcomes of pregnant women. The objective of this study is to measure whether a CHW-oriented mHealth intervention improves the level of knowledge of maternal health of the pregnant women and health-seeking behavior.

In India, the program Mobile for Mothers (MfM) was implemented to strengthen the role of CHWs in improving maternal health outcomes. The project was implemented between 2011 and 2015 in Deoghar block in Jharkhand State, India. MfM was a collaborative effort between regional and international non-governmental organizations (NGOs) including NEEDS (India) and SIMAVI (Netherlands). The accompanying research employed a mixed methods case study design using both qualitative and quantitative tools to assess the effectiveness of MfM among pregnant women. The two target groups of the program were CHWs and pregnant women. The objectives were to (1) improve the delivery of services of CHWs to pregnant women, specifically by providing information and referral services; (2) increase the knowledge and health-seeking behavior of pregnant women in selected rural communities; and (3) improve the collection of key health indicators related to safe motherhood at Block level, which would help district level authorities to better monitor implementation. This study focuses on presenting quantitative results of the evaluation of objective (2)—to increase the knowledge and health-seeing behavior of pregnant women in selected rural communities; the qualitative results are presented in Ilozumba et al. (17).

## Materials and Methods

### Context

According to the recent estimates by the WHO, maternal mortality in India accounts for approximately 15% of global maternal deaths (6). The intervention, MfM was conducted in Jharkhand state, a region with poor maternal health indicators. Jharkhand was 1 of the 18 states explicitly named in the National Rural Health Mission program mission, which addresses the marked differences in health indicators between rural and urban communities, particularly for women and children. A major component of the NHRM was the creation of new category of CHWs, the Accredited Social Health Activists (ASHAs). Due to the lower literacy levels among women in Jharkhand compared with the national level (56 vs. 65%), ASHAs in Jharkhand are not required to have completed class eight education (18). The intervention site, Deoghar block, is a subdistrict of Jharkhand that comprises panchayats and villages. As in Jharkhand state, the majority of the population of Deoghar resides in rural areas (82.6%), and the illiteracy rate is 52% (18). Most recent reports indicate that the percentage of women who received three or more ANC sessions was 48.7%, compared with 60.2% at state level. Institutional delivery was also lower at 39% compared with the state level of 46.2% (18).

### Description of the Intervention

Each ASHA was equipped with an inexpensive Nokia phone that ran free open source software that contains registration forms, checklists, danger sign monitoring, and educational prompts. ASHAs used MfM during home visits to communicate with pregnant women throughout the duration of the program. The mobile application also utilized multimedia functions, meaning that information was presented to the pregnant woman or mother through text, pictures, and also voice prompts. The technological features of the mobile application included the following: (1) voice-based text processions, (2) an alert function when the ASHA should visit the client at home, and (3) the storage of information on the mobile phone. The information was all uploaded to a cloud function managed by a dedicated technology specialist at the local NGO, NEEDS.

### Study Population and Study Design

This was a quasi-experimental cross-sectional study with three groups: (1) an intervention group that received MfM in addition to NGO existing interventions; (2) a quasi-control group that received NGO programs; and (3) a standard care group that only received standard care government programs. All groups received standard care government programs that included the recruitment and support of ASHAs, the Mahatma van scheme, which made ambulances available, and the National Population Stabilization Fund (JSK) scheme, which provided financial incentives for women delivering at the hospital. The quasi-control group (NGO group) in addition to the standard care government offerings also received targeted interventions from NEEDS NGO. These included delivering maternal health education messages among others through dramas and dance, and programs geared at increasing male involvement in maternal health.

Data were collected between Novembers 2015 and January 2016 in Deoghar block. Sample size calculations were conducted with an assumed indicator level of 50% and with a margin of error of ±5% [i.e., the confidence interval (CI) for the coverage estimate is 45–55%] and with a design effect of 1.75 magnitude. This led to a total sample size of 2,200 women, with 740 women per group. Respondents were females between the ages of 18 and 45 currently living in Deoghar, who had delivered in the last 12 months. This study design utilized a multistage cluster sampling approach. Primary Sampling Units, i.e., villages in this case were randomly selected from a list of all villages in each block using a power and sample size analysis. Furthermore, a complete house listing was done to identify the eligible respondents. All households having at least one eligible respondent were included in an Eligible Households Form. The required number of households was selected from the sampling frame through systematic random sampling. In each selected household, one eligible respondent was selected and interviewed.

### Definition of Variables

Interviewers collected the data using a pretested questionnaire. The questionnaire contained all questions in both English and Hindi. The questionnaire consisted of questions related to sociodemographic characteristics: age, age at marriage, age at first delivery, caste [scheduled tribe (ST), scheduled caste (SC), other backward caste (OBC), and other], religion (Hindu, Muslim, Christian, and others), and education level completed of surveyed women and their spouses. Caste was of particular importance, as the role of caste in India has been found significant for maternal health care (19). SCs and STs are on the lowest level of the Indian caste hierarchy and have limited access to educational, social, and economic resources. OBCs are also lower caste but are regarded more favorably than the SC/ST.

This study had three main outcomes related to maternal health: (1) maternal health knowledge, (2) ANC attendance, and (3) delivery at a health facility. Maternal health knowledge was measured across five concepts associated with the main research outcomes, knowledge about the need for: ANC visits, tetanus injections, folic acid supplements, delivery danger signs, and importance about “5 cleans” during delivery. Women were asked how many ANC visits they attended, women were also asked to show their hospital health cards if they had them available. Women reported their delivery location, and for those who reported a facility-based delivery they specified whether they had attended a private hospital or community health center. Trained research assistants who were familiar with the region collected data.

### Statistical Analysis

Primary data were double-entered into Excel spreadsheets by trained research assistants and exported to R software, version 1.0.136 by the researchers (20). All data cleaning and analysis were performed in R. The analysis included three subsets: (1) MfM vs. standard care, (2) MfM vs. NGO, and (3) NGO vs. standard care. The first primary outcome examined was maternal health knowledge, measured as a binary variable where women were considered to have good maternal health knowledge if they answered yes to three of the knowledge questions. The second primary outcome examined was attending ANC services, transformed to a binary factor with women attending four or more ANC, and those who attended three or less. The third primary outcome examined was delivery at a health facility as a binary variable where women either delivered at a health facility or not. Descriptive univariate analyses were performed on the three data subsets. Bivariate analysis was used to investigate the difference in maternal health outcomes among the three different subgroups, and the relationship between maternal health outcomes and individual and household characteristics to evaluate possible confounders. Multivariable logistic regression models were built independently for each of the data sets for each outcome variable. The first model was a full model that contained all predictor variables regardless of their significance in the univariate and bivariate analysis. Variables were then removed in a backward step-wise approach to have a fully adjusted model including all potential predictors. Finally, adjusted odds ratios and 95% CIs were calculated for the effect of the MfM, NGO, and government interventions on maternal health knowledge, ANC attendance, and delivery at a health facility. An alpha level of 0.05 was used to assess statistical significance.

### Ethics

Ethical approval for this study was obtained from the Institutional Review Board-Centre for Media Studies, New Delhi, India. All participating women aged 18–49 provided verbal consent (18). With consideration to the low literacy level among the study population, verbal consent, rather than written or fingerprint documentation was obtained. The consent form was read in Hindi by the interviewer. All participants were informed in detail about the purpose of the study before the start of the interview. They were informed that the data collected were intended for research purposes only and that their data would not be shared with anyone other than essential research team. They were also told that they would not receive any payment for participating and could refuse to answer any questions or stop the survey at any point. No respondents refused to participate in the survey.

## Results

### Characteristics of the Study Populations

Data analysis was conducted independently across the three data subsets: (1) MfM vs. standard care groups (2) MfM vs. NGO groups, and (3) NGO vs. standard care groups. As shown in Table 1, the mean age of the participants across all three groups was 23 years with approximately half of women having 6 years or less of formal education. Approximately two out of three women reported that their spouses had more than 6 years of formal education. There were no significant differences between the three groups on age at marriage and age at first pregnancy. All other variables showed significant differences with regards to sociodemographic characteristics.

**Table 1:** Descriptive characteristics of study population.

Variables	Mobile for Mothers (MfM) (intervention) vs. standard care			MfM (intervention) vs. non-governmental organization (NGO) (quasi-control)			NGO (quasi-control) vs. standard care		
		
	Standard care (*n* = 739)^a^	MfM (*n* = 728)^a^	*P*-values	NGO (*n* = 733)^a^	MfM (*n* = 728)^a^	*P*-values	Standard care (*n* = 739)^a^	NGO (*n* = 733)^a^	*P*-values
**Individual characteristics**									
*Age*									
≤20	153 (20.7)	181 (24.9)	0.06	166 (22.6)	181 (24.9)	0.32	153 (20.7)	166 (22.6)	0.37
21 and older	586 (79.3)	547 (75.1)		567 (77.4)	547 (75.1)		586 (79.3)	567 (77.4)	
*Age at marriage*									
≤18	406 (54.9)	450 (59.1)	0.11	406 (55.4)	430 (59.1)	0.16	406 (54.9)	406 (55.4)	0.86
19–24	333 (45.1)	298 (40.9)		327 (44.6)	298 (40.9)		333 (45.1)	327 (44.6)	
*Age at first pregnancy*									
≤18	304 (41.1)	322 (44.2)	0.23	343 (46.8)	322 (44.2)	0.33	304 (41.1)	343 (46.8)	0.02*
19–24	429 (58.9)	406 (55.8)		390 (53.2)	406 (55.8)		435 (58.9)	390 (53.2)	
*Religion*									
Hindu	610 (82.5)	656 (90.1)	<0.001*	612 (83.5)	656 (90.1)	<0.001*	610 (82.5)	612 (83.5)	0.63
Others (Muslim)	129 (17.5)	72 (9.9)		121 (16.5)	72 (9.9)		129 (17.5)	121 (16.5)	
*Woman’s education*									
Grade 6 or less	417 (56.4)	368 (50.5)	0.02*	414 (56.5)	368 (50.5)	0.07	417 (56.4)	414 (56.5)	0.98
Higher education	322 (43.6)	360 (49.5)		319 (43.5)	360 (49.5)		322 (43.6)	319 (43.5)	

**Household level characteristics**									
*Caste*									
Scheduled caste (SC)/scheduled tribe (ST)	131 (17.7)	201 (27.6)	<0.001*	179 (24.4)	201 (27.6)	0.15	131 (17.7)	179 (24.4)	<0.001*
Non-SC/ST	608 (82.3)	527 (72.4)		554 (75.6)	527 (72.4)		605 (82.3)	554 (75.6)	

**Spouses education**									
Grade 6 or less	332 (44.9)	253 (34.8)	<0.001*	260 (35.5)	253 (34.8)	0.77	332 (44.9)	260 (35.5)	<0.001*
Secondary education	407 (55.1)	475 (65.2)		473 (64.5)	475 (65.2)		407 (55.1)	473 (64.5)	

**Maternal health knowledge**									
0–3	365 (49.4)	210 (28.8)		304 (41.5)	210 (28.8)		365 (49.4)	304 (41.5)	
4–5	374 (50.6)	518 (71.2)	<0.001	429 (58.5)	518 (71.2)	<0.001	374 (50.6)	429 (58.5)	<0.01

**Frequency of antenatal care**									
≥3	611 (82.7)	380 (52.2)	<0.001	534 (72.9)	380 (52.2)	<0.001	611 (82.7)	412 (72.9)	<0.001
≤4	138 (17.3)	348 (47.8)		199 (27.1)	348 (47.8)		128 (17.3)	199 (27.1)	

**Tetanus toxoid vaccine**									
2 doses	291	675	<0.001	527	675	<0.001	292	529	
None or single dose	437	53		201	53		441	204	

**IFA tablets**									
Yes	281	639	<0.001	473	639	<0.001	283	476	<0.001
No	447	89		255	89		450	257	

**Delivery location**									
Non-facility-based delivery	383 (51.8)	163 (22.4)	<0.001	291 (39.7)	163 (22.4)	<0.001		291 (39.7)	<0.001
Facility-based delivery	356 (48.2)	565 (77.6)		442 (60.3)	565 (77.6)		356 (48.2)	442 (60.3)	

*0.05; **0.01; and ***0.001.

*^a^Differences in sample sizes are due to missing data*.

### Bivariate Analysis

At all three subsets, the odds of having a higher score on maternal health knowledge significantly increased when comparing intervention with control (MfM vs. standard care, NGO vs. standard care, and MfM vs. NGO) (see Table 2). In the first subset (MfM vs. standard care), the increase in maternal health knowledge was significantly associated with all independent predictors except religion. In the subset of MfM vs. NGO, the increase in maternal health knowledge was significantly associated with all independent predictors except higher education and religion. Comparisons of caste within each group did not yield any significant results.

**Table 2:** Bivariate associations between independent variables and maternal health knowledge.

	Higher scores in maternal health knowledge					
	Mobile for Mothers (MfM) (intervention) vs. standard subset		MfM (intervention) vs. non-governmental organization (NGO) (quasi-control) subset		NGO (quasi-control) vs. standard subset	
		
	Odds ratio (OR)	95% Confidence interval (CI)	OR	95% CI	OR	95% CI
**Blocks**						
Intervention	1.23	1.17, 1.29***	1.13	1.08, 1.19***	1.08	1.03, 1.14***
Control	1.00 (ref)	–	1.00 (ref)	–	1.00 (ref)	–

**Age (in years)**						
<20	1.08	1.02, 1.15**	1.08	1.02, 1.15***	1.04	0.98, 1.11
>21	1.00 (ref)	–	1.00 (ref)	–	1.00 (ref)	–

**Age at marriage (in years)**						
<18	1.05	1.00, 1.11**	0.95	0.90, 0.99**	1.02	0.97, 1.07
>19	1.00 (ref)	–	1.00 (ref)	–	1.00 (ref)	–

**Education**						
Grade 6 or less	1.00 (ref)	–	1.00 (ref)	–	1.00 (ref)	–
Higher education	1.23	1.17, 1.29***	1.20	1.14, 1.25***	1.26	1.19, 1.32***

**Caste**						
Scheduled caste (SC)/scheduled tribe (ST)	1.00 (ref)	–	1.00 (ref)	–	1.00 (ref)	–
Non-SC/ST	1.08	0.12, 1.15***	1.26	1.19, 1.33***	1.13	1.06, 1.20***

**Religion**						
Hindu	1.00 (ref)	–	1.00 (ref)	–	1.00 (ref)	–
Other religions	1.04	0.97, 1.12	0.99	0.92, 1.06	0.98	0.92, 1.05

**Spouses education**						
Grade 6 or less	1.00 (ref)	–	1.00 (ref)	–	1.00 (ref)	–
Higher education	1.33	1.26, 1.39***	1.22	1.15, 1.28	1.29	1.23, 1.36***

*0.05; **0.01; and ***0.001.

Women in the MfM group were more likely to attend four or more ANC visits than those in the standard care group [odds ratio (OR), 1.36; 95% CI, 1.30, 1.42] and those in the NGO group (OR, 1.23; 95% CI, 1.17, 1.29) (Table 3). In addition, those in the NGO group also had higher odds of attending four or more ANC visits than the standard care group (OR, 1.10; 95% CI, 1.06, 1.15). At all subsets, women who belonged to a non-SC/ST caste were significantly more likely to attend ANC compared with women within SC/ST caste. At two subsets (MfM vs. standard care and MfM vs. NGO), women younger than 20 were more likely to attend ANC compared with those older than 20. At two subsets (MfM vs. NGO and NGO vs. standard care), women with higher educational levels and a spouse with higher education were more likely to utilize ANC services compared with women with lower educational levels and a spouse with lower education, respectively.

**Table 3:** Bivariate analysis: association between individual and household characteristics as covariates, group as a primary study variable and ANC attendance outcome.

	Attending 4 or more antenatal care visits					
	Mobile for Mothers (MfM) (intervention) vs. standard subset		MfM (intervention) vs. non-governmental organization (NGO) (quasi-control) subset		NGO (quasi-control) vs. standard subset	
		
	Odds ratio (OR)	95% Confidence interval (CI)	OR	95% CI	OR	95% CI
**Blocks**						
Intervention	1.36	1.30, 1.42***	1.23	1.17, 1.29***	1.10	1.06, 1.15***
Control	**ref**					

**Age (in years)**						
<20	1.06	1.00, 1.12**	1.07	0.96, 1.06	1.04	0.99, 1.11
>21	1.00 (ref)	–	1.00 (ref)	–	1.00 (ref)	–

**Age at marriage (in years)**						
<18	1.07	1.02, 1.12**	1.01	-0.04, 0.06	0.98	0.94, 1.02
>19	1.00 (ref)	–	1.00 (ref)	–	1.00 (ref)	–

**Education**						
Grade 6 or less	1.00 (ref)	–	1.00 (ref)	–	1.00 (ref)	–
Higher education	0.98	0.94, 1.03	1.09	1.03, 1.15***	1.05	1.01, 1.10**

**Caste**						
Scheduled caste (SC)/scheduled tribe (ST)	1.00 (ref)	–	1.00 (ref)	–	1.00 (ref)	–
Non-SC/ST	1.05	1.00, 1.11*	1.11	1.05, 1.17***	1.13	1.07, 1.19***

**Religion**						
Hindu	1.00 (ref)	–	1.00 (ref)	–	1.00 (ref)	–
Other religions	0.92	0.86, 0.98	1.14	1.06, 1.23	1.02	0.96, 1.08

**Spouses education**						
Grade 6 or less	1.00 (ref)	–	1.00 (ref)	–	1.00 (ref)	–
Higher education	1.01	0.97, 1.06	1.09	1.03, 1.15***	1.17	1.11, 1.23***

*0.05; **0.01; and ***0.001.

*The bold fonts indicate higher level categories*.

The odds of a woman in the MfM group delivering at a health center were significantly higher than the odds of women in the standard care group (OR, 1.34 95% CI, 1.28, 1.41) and NGO group (OR, 1.19; 95% CI, 1.13, 1.25) (Table 4). At all three subsets, women getting married before the age of 18, having higher education and belonging to a non-SC/ST tribe were significantly associated with delivering at a health facility compared with women getting married after the age of 18, having lower education, and belonging to an SC/ST tribe. In the MfM vs. NGO subset, religion (OR, 1.13, 95% CI, 1.07, 1.19) and education of the spouse (OR, 1.17, 95% CI, 1.12, 1.23) were also significantly associated with higher odds of women to deliver at a health facility.

**Table 4:** Bivariate analysis: association between individual and household characteristics as covariates, group as a primary study variable and delivery location outcome.

	Delivering at a health facility					
	Mobile for Mothers (MfM) (intervention) vs. standard subset		MfM (intervention) vs. non-governmental organization (NGO) (quasi-control) subset		NGO (quasi-control) vs. standard subset	
		
	Odds ratio (OR)	95% Confidence interval (CI)	OR	95% CI	OR	95% CI
**Blocks**						
Intervention	1.34	1.28, 1.41***	1.19	1.13, 1.25	1.13	1.07, 1.19
Control	**ref**					

**Age (in years)**						
<19	1.05	0.98, 1.11	1.07	1.02, 1.14**	1.03	0.97, 1.10
>35	1.00 (ref)	–	1.00 (ref)	–	1.00 (ref)	–

**Age at marriage (in years)**						
<18	0.95	0.91, 1.00*	0.93	0.89, 0.97***	0.91	0.87, 0.96***
>19	1.00 (ref)	–	1.00 (ref)	–	1.00 (ref)	–

**Education**						
Grade 6 or less	1.00 (ref)	–	1.00 (ref)	–	1.00 (ref)	–
Higher education	1.16	1.11, 1.22***	1.20	1.15, 1.26***	1.19	1.13, 1.25***

**Caste**						
Scheduled caste (SC)/scheduled tribe (ST)	1.00 (ref)	–	1.00 (ref)	–	1.00 (ref)	–
Non-SC/ST	1.12	1.05, 1.19***	1.13	1.07, 1.19***	1.17	1.10, 1.24***

**Religion**						
Hindu	1.00 (ref)	–	1.00 (ref)	–	1.00 (ref)	–
Other religions	1.04	0.96, 1.11	1.15	1.07, 1.23***	1.03	0.96, 1.10

**Spouses education**						
Grade 6 or less	1.00 (ref)	–	1.00 (ref)	–	1.00 (ref)	–
Higher education	1.14	1.09, 1.20	1.17	1.12, 1.23***	1.17	1.11, 1.23***

*0.05; **0.01; and ***0.001.

*The bold fonts indicate higher level categories*.

### Multivariable Analysis

Although multivariable models were built independently for the three main outcomes comparing the three-analysis groups, there were similarities in the final models across groups. Across all three groups, higher maternal health knowledge, ANC attendance, and delivery at a health facility were most strongly associated with belonging in the intervention group (MfM and NGO).

At all three subsets, the adjusted odds of having a higher score on maternal health knowledge significantly increased with for types of interventions (NGO and MfM) when adjusting for the woman’s education level, her caste and her spouse’s education level and caste (MfM vs. NGO: OR, 1.14, 95% CI, 1.08, 1.19). For the MfM vs. standard care group, belonging to the intervention group, age at marriage, education, and spouses education were the most significant predictors of higher maternal health score (OR, 1.19, 95% CI, 1.13, 1.25).

Belonging to a non-SC/ST caste, belonging to a religion other than Hindu and being in the intervention group were the most significant predictors of attending ANC both against the NGO (OR, 1.22, 95% CI, 1.17, 1.28) and standard care group (OR, 1.38, 95% CI, 1.32, 1.44). The final model for all three groups regarding delivering at a health facility was the same, with belonging to the intervention group (MfM or NGO), having a higher level of education and belonging to a caste other than SC/ST being the most significant predictors of delivering at a health facility (Table 5).

**Table 5:** Backward step-wise multivariable logistic regression analysis of significant factor association with higher maternal health knowledge, attending four or more antenatal care (ANC) visits, and delivering at a health facility among members of the three subsets.

	Mobile for Mothers (MfM) (intervention) vs. standard care subset Adjusted odds ratio (AOR) [95% confidence interval (CI)]	MfM (intervention) vs. non-governmental organization (NGO) (quasi-control) subset AOR (95% CI)	NGO (quasi-control) vs. standard care subset AOR (95% CI)
**Higher maternal health knowledge**			
Intervention	1.19 (1.13, 1.25)	1.14 (1.08, 1.19)	1.06 (1.01, 1.12)
Control	**1.00 (ref)**	1.00 (ref)	1.00 (ref)
Adjusted for	Age at marriage, education, and spouses education	Education, caste, and spouses education	Age at marriage, education, and spouses education

**Attended 4 or more ANC attendance**			
Intervention	1.38 (1.32, 1.44)	1.22 (1.17, 1.28)	1.11 (1.06, 1.16)
Control	1.00 (ref)	1.00 (ref)	1.00 (ref)
Adjusted for	Caste and religion	Caste and religion	Caste

**Delivered at a health facility**			
Intervention	1.35 (1.29, 1.42)	1.18 (1.12, 1.24)	1.14 (1.08, 1.20)
Control			
Adjusted for	Caste and education	Caste and education	Caste and education

*The bold fonts indicate higher level categories*.

## Discussion

This study showed that women in our MfM intervention group reported higher levels of maternal health knowledge than those in the NGO intervention or who received standard care. This is important because despite the growing number of mHealth programs utilized by CHW, there are a limited number of interventions examining the effects of such interventions on maternal health outcomes. The majority of the work on maternal health outcomes is focused on mHealth programs that target the pregnant woman as an end user (16).

Maternal health knowledge in our study was measured as women reporting that they understood why they should receive specific types of maternal health services. While this result is encouraging, past research shows that maternal health knowledge is not directly correlated with maternal health practice (21). Maternal health knowledge is, however, the first step in a complex process of improving maternal health outcomes. Women’s understanding of specific maternal health concepts, such as the need for proper nutrition and rest, hygiene during delivery, and danger signs, is essential in the prevention of maternal health complications (22).

In our study, women in the MfM intervention group were more likely to attend four or more ANC visits before delivery than women in the NGO or standard care group. It is important to note that while this finding is positive, overall ANC attendance in our study remained low, with the MfM group reporting about one in two women attending four or more ANC sessions. However, in the NGO and standard care groups only about one in four women had four or more ANC visits. These numbers remain suboptimal when compared with international recommendations (6). It is important to note that while the most recent WHO recommendations have set eight ANC visits as the standard care, in countries like India achieving 100% attendance of four ANC visits by pregnant women has proved elusive.

While the MfM intervention group showed a higher likelihood of delivering in a health facility, approximately 50% of all participants reported utilizing health-care facilities for delivery. This finding highlights the importance of contextual factors in improving pregnant women’s utilization of services. While the mobile application provides the necessary knowledge to ensure women are informed about the need to deliver at the health facility it does not address factors such as women’s autonomy or health system constraints (23, 24). The study area, like many rural parts of India and other LMICs, has a limited number of health facilities that are frequently situated too far away from communities. Shortages of necessary health professionals, equipment, and drugs are not uncommon. Past research also shows that bribes are frequently requested from women and their families at health facilities. These factors and others, which ultimately contribute to low levels of facility-based delivery, cannot be overcome by mHealth technologies, such as MfM.

After adjusting for sociodemographic factors like age of the mother, educational level of the father, other than belonging to an intervention group (MfM or NGO) caste was the most reoccurring predictor of maternal health knowledge, attending ANC and delivering at a health facility. The significance of caste to maternal health outcomes has been highlighted in past research. One study found that women who were Muslim of non-STs were more likely to receive ANC (14). In our study, belonging to a no SC/ST caste and not being Hindu were significantly correlated with delivering at a health facility. Other studies, including statewide analysis revealed that while lower caste was correlated to delivery at a health facility in one state, in other states it was associated with poorer maternal health service utilization (16). The variations in maternal service utilization based on caste and the impact of social exclusion remain under-explored in health research. The caste system in India is entrenched and has remained resistant to change. There are reports of disrespectful childbirth and treatment in general by health professionals for women belonging to SC or tribes (25, 26).

Age while significant in bivariate analysis was not correlated to any of the health outcomes when other factors were controlled for. In India, there is evidence that women between the ages of 25 and 34 utilize ANC at higher rates than other age groups (15). Our study does not report similar findings, probably due to the fact that the average age of the women surveyed was 23 years old. This relatively homogenous age distribution could be related to our finding that that being younger than 20 years old was correlated with better ANC attendance. However, our findings were in line with prior research that found women younger than 24 were more likely to deliver their babies at health facilities as compared with other age groups (15). However, when we controlled for other variables, age was not significantly associated with delivering at a health facility. Delivering at health facility has been related to parity, that is, the number of children increases the utilization of health facilities for delivery tends to decrease (15). Considering the average age of marriage in our sample was 17 years old, and the average age at interview time was 23 years old, our sample can reasonably be expected to have lower parity.

Our results provide an interesting counterpoint to prior research that highlighted that disenfranchised women (including women who lived in rural areas, belonging to lower social economic class, and were illiterate) may have less access to mHealth (15). These studies mostly focused on intervention where women directly received text messages from a central source. In such a situation, the ability of a woman to operate the mobile phone and read the text message becomes more significant. This study investigated outcomes on women who were in similar rural and resource-poor settings. Yet, we find that mHealth still had a significant effect. We suggest that the utilization of the mHealth application by CHWs could have been an important factor—the CHWs served as an intermediary to the understanding of the mother. This could be because the existing knowledge and training of the CHW would enable them to use the mobile application more easily than a woman in their community. Another possibility could be that the women received the messages in the presence of the CHW, providing opportunities for questioning and clarification. Although more research is needed, we believe that our study results suggest that the CHW as an end user of the intervention could have a positive effect on maternal health outcomes.

### Limitations

This study answers an important question about the future of mHealth interventions for maternal health. There are, however, limitations related to study design and methodology. We were unable to conduct pre- and post-intervention analysis. Although a baseline household survey was conducted at the start of the intervention, repeated measures were not performed on the same individuals making the data unusable for complex analysis. This has possible implications for the study results presented in this study, including the possibility that although the groups were similar on sociodemographics, there might have already been differences in the outcome measures of maternal health knowledge; ANC attendance and delivery at health facilities at baseline. However, the comparatively large sample size for an mHealth study and the quasi-experimental design lend credence to the validity of our results. There were also additional limitations in regards to the omission of potentially relevant indicators, as relative distance of health service centers and parity was not collected. However, the three geographical locations for the study were also chosen based on their similarity with respect to sociodemographics and access to health-care facilities. In addition, the survey instrument was created in consultation with experts and pilot tested before use; however, it was not validated. This lack of validation is particularly related to the accuracy of the maternal health knowledge variable as a sufficient measure of true knowledge.

### Implications for Research/Practice/Policy

In the last couple of years, more studies are emerging which show the effect of mHealth on multiple aspects of maternal health outcomes. This study contributes to that by providing evidence that mHealth can lead to better uptake of maternal health services. However, the study results also showed that the NGO programs which were not mHealth based were also correlated to greater likelihood of ANC attendance and delivery at a health facility, albeit at a lower rate.

It is important for future research to consider not only sociodemographic characteristics of women and family in relation to maternal health outcomes but also those of the CHWs. In a setting like India, where home visits by CHWs are an intervention on their own, and in our case were the NGO provided additional interventions, small-scale theater, male engagement meetings, and training of CHWs, a question of attribution arises. Future research needs to ask questions about the specific contributions of the mHealth interventions. Related to this question is the need for cost effectiveness studies on the utilization of mHealth over none technological maternal health interventions. Further research, particularly qualitative, is needed to understand external factors that impact the results of similar mHealth interventions.

## Conclusion

Our results show that mHealth had a significant effect on higher levels of maternal health knowledge, increased attendance of ANC and delivering at a health facility among the participants. The MfM intervention led to statistically significant differences in maternal health knowledge, ANC attendance, and delivery at a health institution. The evidence from this study makes a strong case for the application of similar mHealth interventions in LMICs. However, the strong influence of caste on all three of these maternal health outcomes suggests the importance of cultural context in the development of interventions. CHW utilized mHealth interventions can be effective at circumventing the effects of maternal literacy; however, they operate within a sociocultural context, that must be taken into account in program design.

## Ethics Statement

Ethical approval for this study was obtained from the Institutional Review Board-Center for Media Studies, New Delhi, India. With consideration to the low literacy level among the study population, verbal consent, rather than documentation was obtained. The consent form was read in Hindi by the interviewer. All participants were informed in detail about the purpose of the study before the start of the interview. They were informed that their participation was voluntary and that they had the right to skip questions they felt uncomfortable with or stop answering at any time. Identifying information such as names or addresses was not collected to protect participant confidentiality. There were no psychological or social risks involved in participation in this study. They were informed that the data collected were intended for research purposes only and that their data would not be shared with anyone other than essential research team.

## Author Contributions

Conceptualized the paper, searched literature, performed statistical analysis, and drafted the manuscript: OI. Intervention conception and design: LL and MC. Made critical revisions to the paper: OI, SVB, MD, LL, MC, and JB. Supervised the study: SVB, MD, and JB. All the authors read and approved the final manuscript.

## Conflict of Interest Statement

The authors declare that the research was conducted in the absence of any commercial or financial relationships that could be construed as a potential conflict of interest.

## Abbreviations

ANC, antenatal care; AOR, adjusted odds ratio; ASHA, Accredited Social Health Activist; CHWs, community health workers; CI, confidence interval; LMICs, low- and middle-income countries; mHealth, mobile health; NGO, non-governmental organization; NRHM, National Rural Health Mission; OBC, other backward caste; OR, odds ratio; PDAs, personal digital assistants; MfM, Mobile for Mothers; PSS, power and sample size; PSU, Primary Sampling Units; SC, scheduled caste; ST, scheduled tribe; WHO, World Health Organization.
